# *Saussurea costus* extract as bio mediator in synthesis iron oxide nanoparticles (IONPs) and their antimicrobial ability

**DOI:** 10.1371/journal.pone.0282443

**Published:** 2023-03-09

**Authors:** Jehan S. Al-brahim

**Affiliations:** Department of Biology, College of Sciences, Princess Nourah bint Abdulrahman University, Riyadh, Saudi Arabia; King Abdulaziz University, SAUDI ARABIA

## Abstract

*Saussurea costus* is from medicinal plants and have therapeutic properties that were recorded in a variety of medical functions. The usage of biomaterials in the synthesis of nanoparticles is an essential strategy in green nanotechnology. Iron oxide nanoparticles (IONPs) were composed in the stage of (2:1, FeCl_2_: FeCl_3_) solution by using the aqueous extract of *Saussurea costus* peel in an eco-friendly method to evaluate their antimicrobial property. The properties of the obtained IONPs were evaluated using a scanning (SEM) and transmission (TEM) electron microscope. The mean size of IONPs discovered by Zetasizer varies between 100 and 300 nm, with a mean particle size of 295 nm. The morphology of IONPs (γ-Fe_2_O_3_) was determined to be nearly spherical and prismatic-curved. Moreover, the antimicrobial property of IONPs was assessed with nine pathogenic microbes, revealing that the nanoparticles have antimicrobial activities with *Pseudomonas aeruginosa*, *Escherichia coli*, *Shigella* sp., *Staphylococcus* sp. and *Aspergillus niger*, with possible applications in the therapeutic and biomedical fields.

## Introduction

Plant-derived medicines continue as a valuable resource, particularly in developing countries, for treating severe ailments. Around 60–80 percent of people worldwide mostly use folk medicine in treating diseases [1]. A wide range of biological activities, including antimicrobial properties, have been demonstrated in medicinal plant extracts [2]. Medicinal plants include a variety of phytochemicals [1, 3]. All those substances, including tannins, terpenoids, alkaloids, and flavonoids, that have antimicrobial properties [4]. Plants have long been used in treating human wellness issues with therapeutic medication in multiple historical systems. The cosmetics, dairy, color, and pharmaceutical industries frequently employ natural plant extracts due to their potential medicinal and pharmaceutical applications [5].

*Saussurea costus*, (also known as *Saussurea lappa*) also identified as "Al-Kost Al-Hindi" in Arab countries, belongs to the Asteraceae family, which has many species in India, Pakistan, and parts of the Himalayas [6]. *S*. *costus’s* therapeutic properties were recorded in a variety of medical functions. For centuries, several people have used the fragrant roots of *S*. *costus* to make medications, incense, and ointments [7]. Traditionally, *S*. *costus* has been used as a psychoactive, antibiotic, repellent, anesthetic, and antispasmodic [8]. Numerous studies have found that *S*. *costus* roots possess antiulcer, anti-inflammatory, anti-immune, and antiviral activity [9].

Functional nanoscale particle have greater potential for building projects and the advancement of nanotechnology than bulk materials [10, 11]. Lately, one of the best ways to combat antimicrobial resistance in bacteria is to use nano-technological solutions like nanoparticles [12–15]. Nanoparticles (NPs) have antimicrobial properties with gram-negative and gram-positive bacteria [16–18]. Nanoparticles also enhance plant growth and yield when used correctly [19]. Plant are utilized in the formation of nanoparticles as a reduction agent, which reduces the use of toxic-reducing substances [20]. Iron is one of the most widely available elements on earth [21]. At the same time, iron is required for the spread of infection by microbes that have evolved methods of acquiring iron from the host, whilst the host has defense functions that protect microbes from obtaining iron [22]. IONPs have applications in medical sciences and other industries, including gas sensors, electrochemical, magnetic, and energy storage [23]. Iron oxide nanoparticles are magnetic as well as paramagnetic [24]. The iron oxide NPs superparamagnetic behavior has led to their use in imaging, drug delivery, targeting, and biosensors. Besides that, their distinct properties, like biocompatibility, strong magnetic properties, low toxicity, and catalytic behavior, have aided to biomedical applications [23]. The IONPs have antimicrobial properties [25]. Both gram-positive and gram-negative bacteria are resistant to IONPs [26]. Various studies on the eco-friendly creation of IONPs were conducted utilizing various plant parts to assess their bioactivity, IONPs synthesized using *Couroupita guianensis* fruit extract had effective bactericidal against tested human pathogens [27]. IONPs derived from corn plant extract displayed antibacterial activity against the tested bacterial species [24].

The research aimed to synthesize IONPs utilize the dried root bark extract of Saudi *Saussurea costus* as a reductant in the eco-friendly method, incorporating the therapeutic properties of *S*. *costus* root extract with the features of IONPs and assessing their antimicrobial efficiency with three fungi and six bacteria. This study is first Saudi Arabian study entitled “Synthesis of iron oxide nanoparticles from *Saussurea* extract”, and it was granted a patent number (SA 10300), (IPK^8^): A61K 036/000.

## Materials and methods

### Iron oxide nanoparticles formation

**Fig 1:**
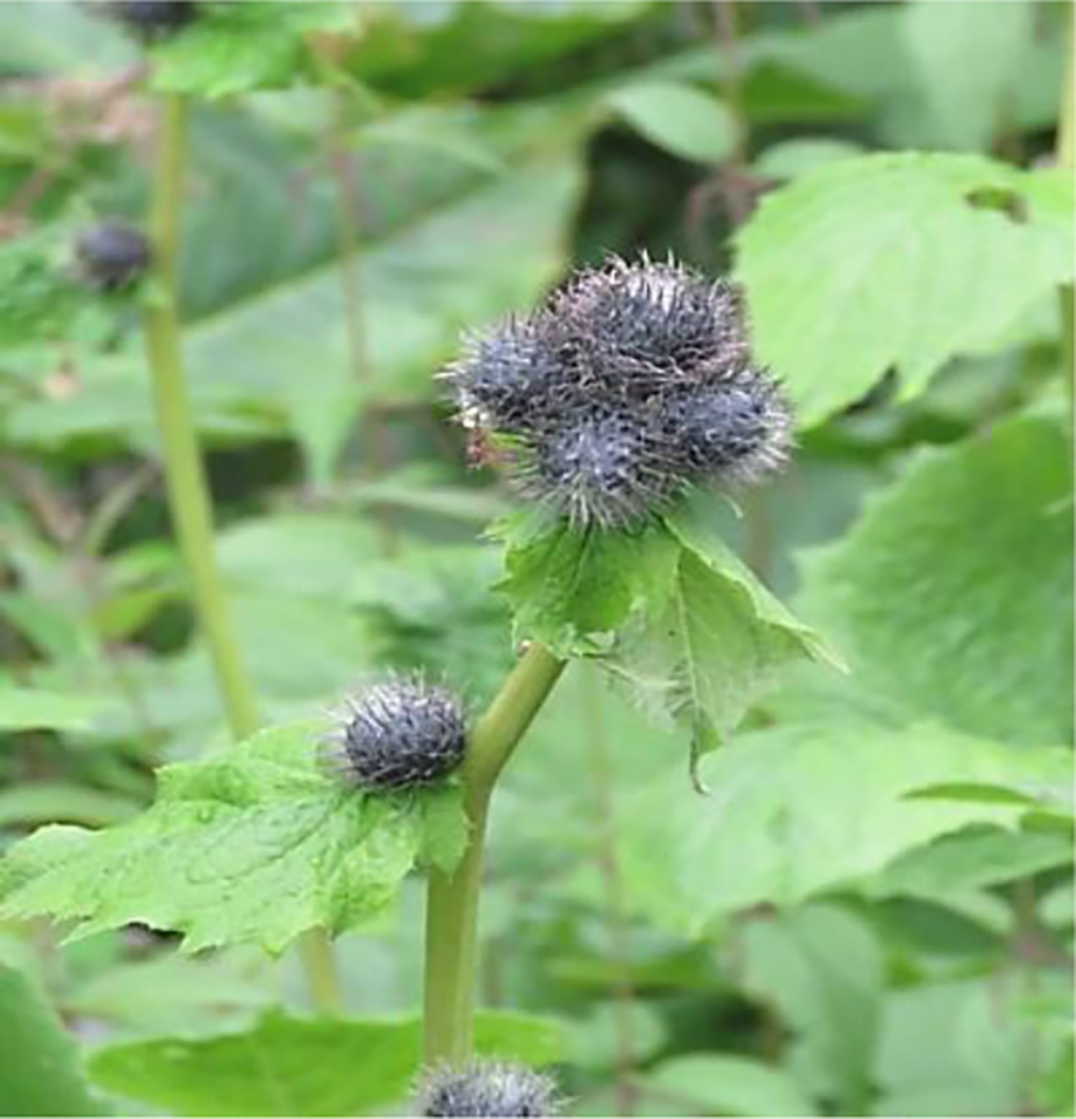
The figure shows *Saussurea costus* plant.

*Saussurea costus* peel specimens were bought from a local market in Riyadh, Saudi Arabia (Fig 1) [28]. The dry *S*. *costus* root peel was fermented and dried with water. A milling machine was used to grind dehydrated specimens into powder form. Extracts were produced from 20 g of plant powder in 100 ml of 50% methanol and stirred with a magnetic stirrer until a homogeneous solution was formed. To prevent enzyme activity, the aqueous extract was heated for 30 min at 70°C in a water bath. Furthermore, the extract was filtered over the Whatman mentioned above. In a simple method, iron oxide NPs were formed by adding 100 ml of onion peel extract into (50 mL of 1 mM FeCl_2_+ 100 mL of 1 mM FeCl_3_). To concentrate the extract, 5 ml of the prepared peel extract was blended with 45 ml (2: 1, FeCl_2_: FeCl_3_) solution in a flask and left at room temperature for 24 hours. Then, a magnetic stirrer was used to agitate the solution for 30 minutes at 80°C. The first indicator of γ-Fe_2_O_3_ NPs production as a result of reducing iron ions to iron nanoparticles is the exchange of the binder color from yellow to dark brown. The use was kept at 4°C for further IONP description and apps [29].

### Microbial strains

We utilized 6 bacterial and 3 fungal strains, including *Staphylococcus sp*., *Bacillus subtilis* gram-positive, *Klebsiella pneumonia*, *Shigella* sp., *Pseudomonas aeruginosa*, *Escherichia coli* gram-negative, and *Aspergillus niger*, *Alternaria sp*., and *Rhizopus* sp. Fungi, obtained from the biology department faculty of sciences, university of princess Nourha bint Abdulrahman (Table 1).

**Table 1:** The antimicrobial properties of (IONPs) obtained from *S*. *costus* root extract; the table displays the diameter of the inhibition zone (mm) around the hole.

	Bacterial strain	NPs (mm)	Fe ions (mm)	Plant extract (mm)
**A**	***Staphylococcus sp*.**	**9**	**1.8**	**7**
**B**	***Klebsiella pneumonia***	**-**	**1.8**	**-**
**C**	***Shigella sp*.**	**6**	**1.7**	**-**
**D**	***Bacillus subtilis***	**-**	**-**	**-**
**E**	***Pseudomonas aeruginosa***	**11**	**1.7**	**8**
**F**	***Escherichia coli***	**9**	**1.7**	**8**
**G**	***Aspergillus niger***	**5**	**1.1**	**-**
**H**	***Alternaria sp*.**	**-**	**-**	**-**
**I**	***Rhizopus sp*.**	**-**	**-**	**-**

### Identification and characterization of IONPs

The characterizations of the bioactive IONPs were investigated and used a UV-2450 double-beam (200–800 nm) UV-spectrophotometer (Shimadzu, Tokyo, Japan). The size and morphology of biogenic IONPs were determined using a transmission electron microscope (TEM), a JEOL JEM-1011 (JEOL, Peabody, MA, USA), and field emission scanning electron microscopy (JEOL 7500FA JEOL, Peabody, MA, USA) at 200 kV and 30 kV voltage, respectively. A zeta size tool was used to assess the average size distribution and the zeta potential (Malvern, Worcestershire, UK).

### Antimicrobial properties

The anti-microbial efficient acts of iron nanoparticles were calculated using a well-gar dispersion strategy. They were cultured on Mueller-Hinton Agar at a concentration of 108/ml for bacterial growth and potato agar for fungal growth. Three fungi forms, *Aspergillus niger*, *Alternaria* sp., *Rhizopus* sp., and bacteria forms, the gram-positive *Staphylococcus sp*., *Bacillus subtilis*, and gram-negative community *Klebsiella pneumonia*, *Shigella* sp., *Pseudomonas aeruginosa*, and *Escherichia coli*, have been examined (Table 1). To measure antimicrobial efficiency, 100 μL of biosynthesized iron oxide nanoparticles and 100 μL of water were added as a negative agent. Bacterial strains were isolated in 0.2 mL (CFU 2.5*10^5^ mL). On every agar plate, sterile swabs are placed at irregular intervals. Three of them were properly separated. The sterile cultivation agar surface was used to create wells (holes) with a diameter of 4 mm per plate. Use a metal cork to bore the hole. The aseptic use of 0.2 mL of extract at every hole was performed in the room. The excerpts can be dispersed and cultivated in the agar medium after 1 hour at room temperature. The negative control used in comparison was aseptic pure water. The plates were kept for 24–48 hours at 37°C and 25°C. Inhibition zones were identified as distinct regions surrounding the pools [30].

### Investigation of bacterial cell membrane

Treated bacteria were examined using FE-SEM to observe any modifications in the bacterial shape and to identify the exact mechanisms of action of IONPs with each examined microbe. IONPs from *S*. *costus* were added to the bacterial growth at MIC levels, and the cultures were kept at 37°C for 1 hour. Treated cells were centrifuged at 6000 rpm for 10 minutes at 4°C. PBS was used to wash the pellets at pH 7, and they were then fixed for 1 h in glutaraldehyde 8% and Sorensen’s phosphate buffer (SPB). To fix the samples, 4% osmium tetroxide (OsO4) and H_2_O (1:1) volume were mixed. The specimen was examined using FE-SEM (JEOL JSM-7001F, Germany) after it had been washed and dehydrated for 24 hours.

## Results

### Morphology and characters of biogenic IONPs

*Saussurea costus* peel extract were used a biosynthetic approach to generate IONPs from mineral solutions with a concentration of 1 mM. The iron oxide nanoparticles (Fe_3_O_4_) produced by using a mixture of (2:1, FeCl_2:_ FeCl_3_) and *S*. *costus* root as a reducing agent resulted in a dark brown color of the solution, which had previously been yellow. The color of the combination changed in a time-dependent manner for 24 hours at dark room temp. As an indicator of iron oxide formation, the dark brown color was steady and evident.

**Fig 2:**
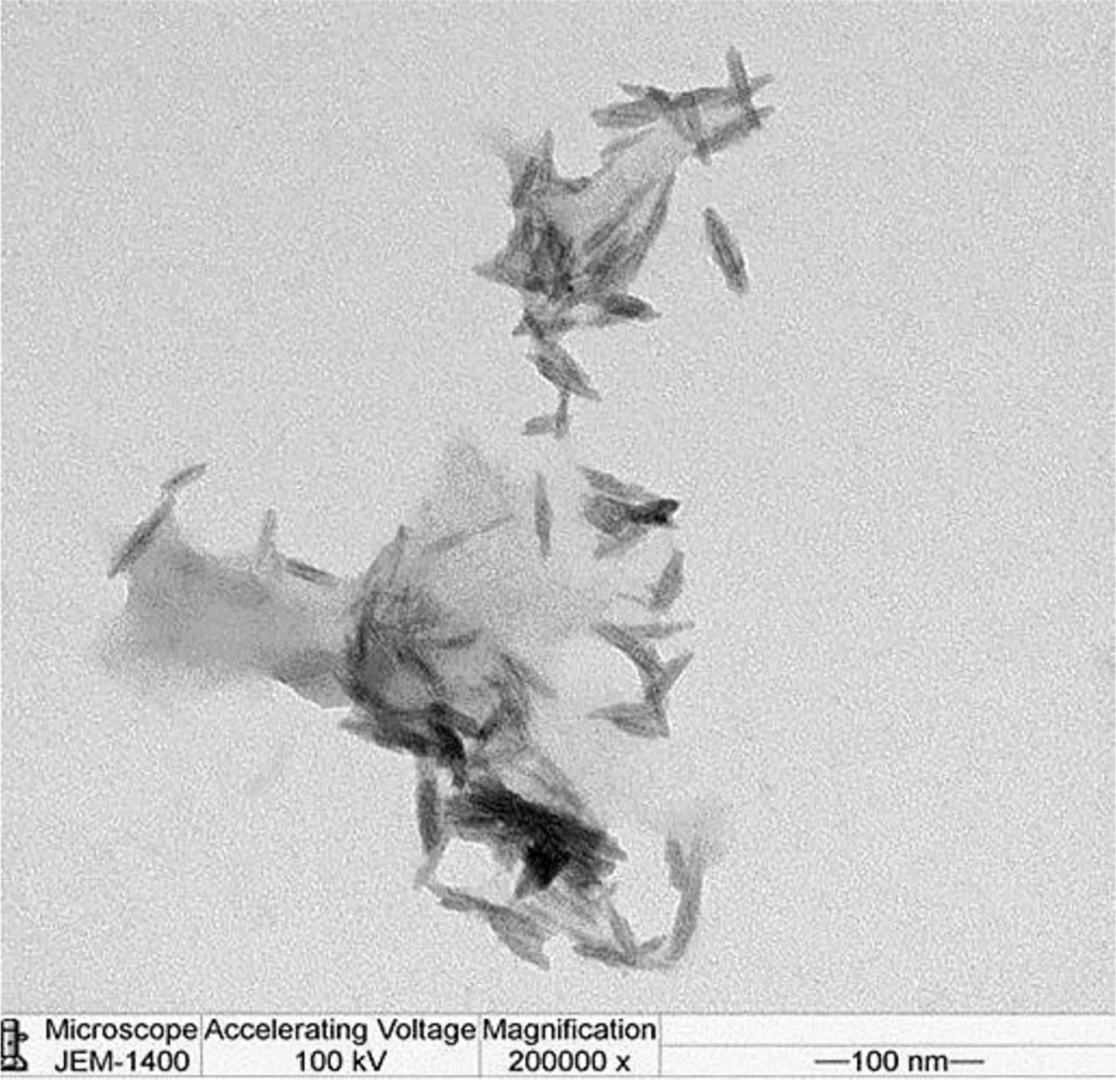
TEM images of (IONPs) obtained using *S*. *costus* root extracts. Magnification is 200,000 and scale bar represents 1 and 100 nm.

**Fig 3:**
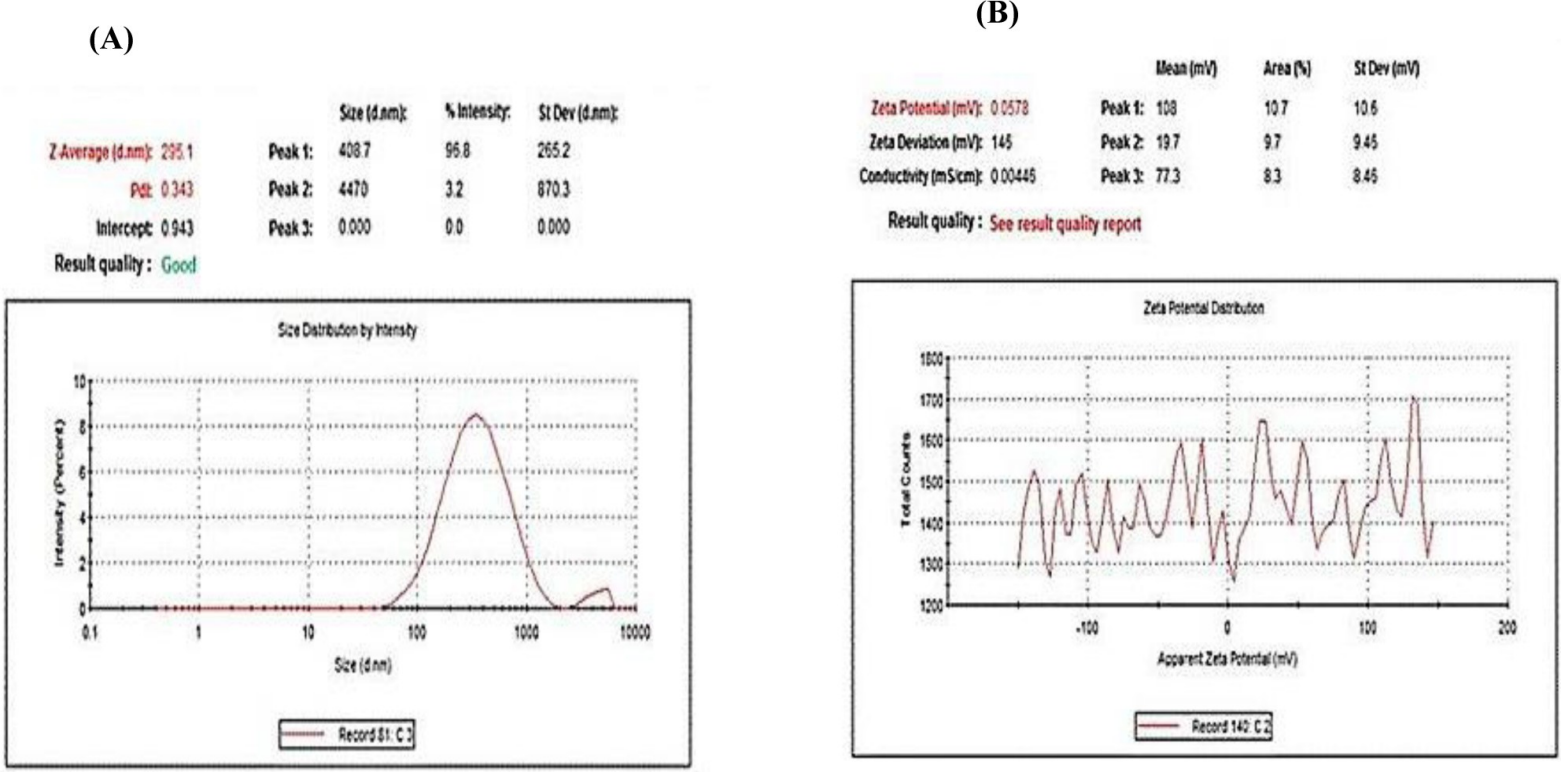
Zeta size images shows (A) the average particle size and (B) the potential of the synthesized (IONPs) from *S*. *costus* root extracts.

**Fig 4:**
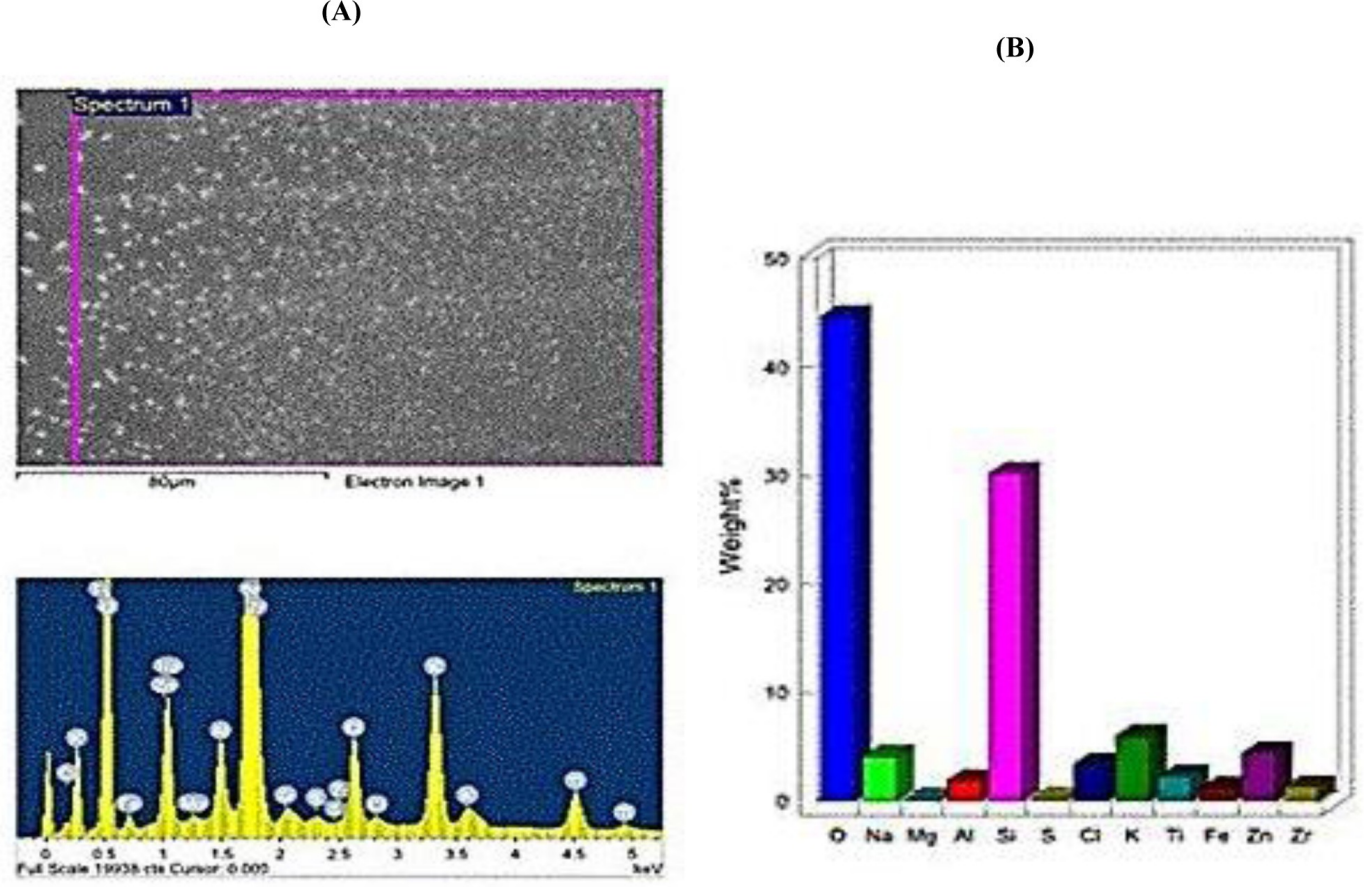
SEM-EDX images (A) and the corresponding element analysis images (B) of (IONPs) obtained from *S*. *costus* root extracts.

SEM and TEM photos, in contrast, were employed to investigate the morphology, size, and surface forms of biogenic IONPs. IONPs were to be spherical, sided, elongated, spherical, and prismatic curve-shaped when TEM and SEM were used (Figs 2 and 4A). The diameter of particle size for IONPs was varied from 100 to 300 nm, with an average of 295 nm, by using Zetasizer (Fig 3A). The positive potential of IONPs particles was produced from *S*. *costus* roots by using Zetasizer (Fig 3B). The particles’ stability was demonstrated by the presence of a zeta potential in the +0.058 mV range. The element analysis of the biogenic IONPs prepared using SEM-EDX (Fig 4B) revealed the presence of various elements, with oxygen and silicon being the main components. In addition to Fe, other elemental peak bands for O, Na, Mg, Al, Si, S, Cl, K, Ti, Zn, and Zr were detected through the spectral scan range (0–5 keV) (Fig 4A).

### The antimicrobial property of the IONPs

**Fig 5:**
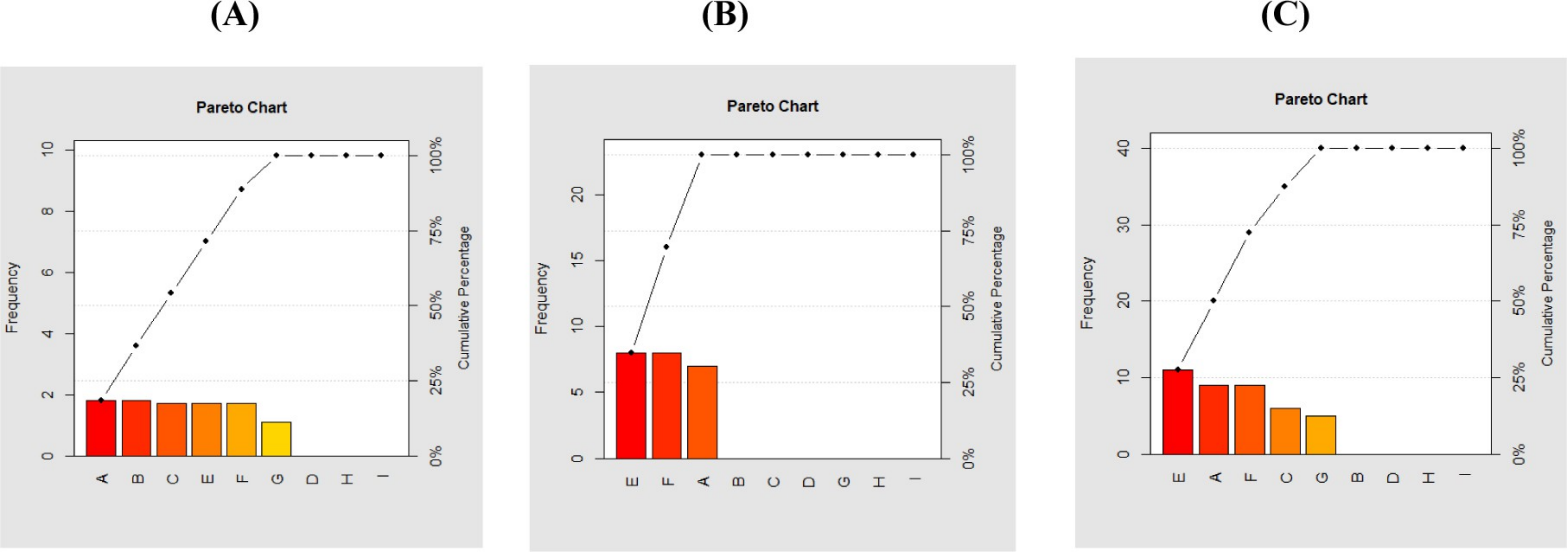
The above chart output displays the frequency and cumulative frequency of each (A) NPs; (B) Fe. Ions and (C) Plants.

**Fig 6:**
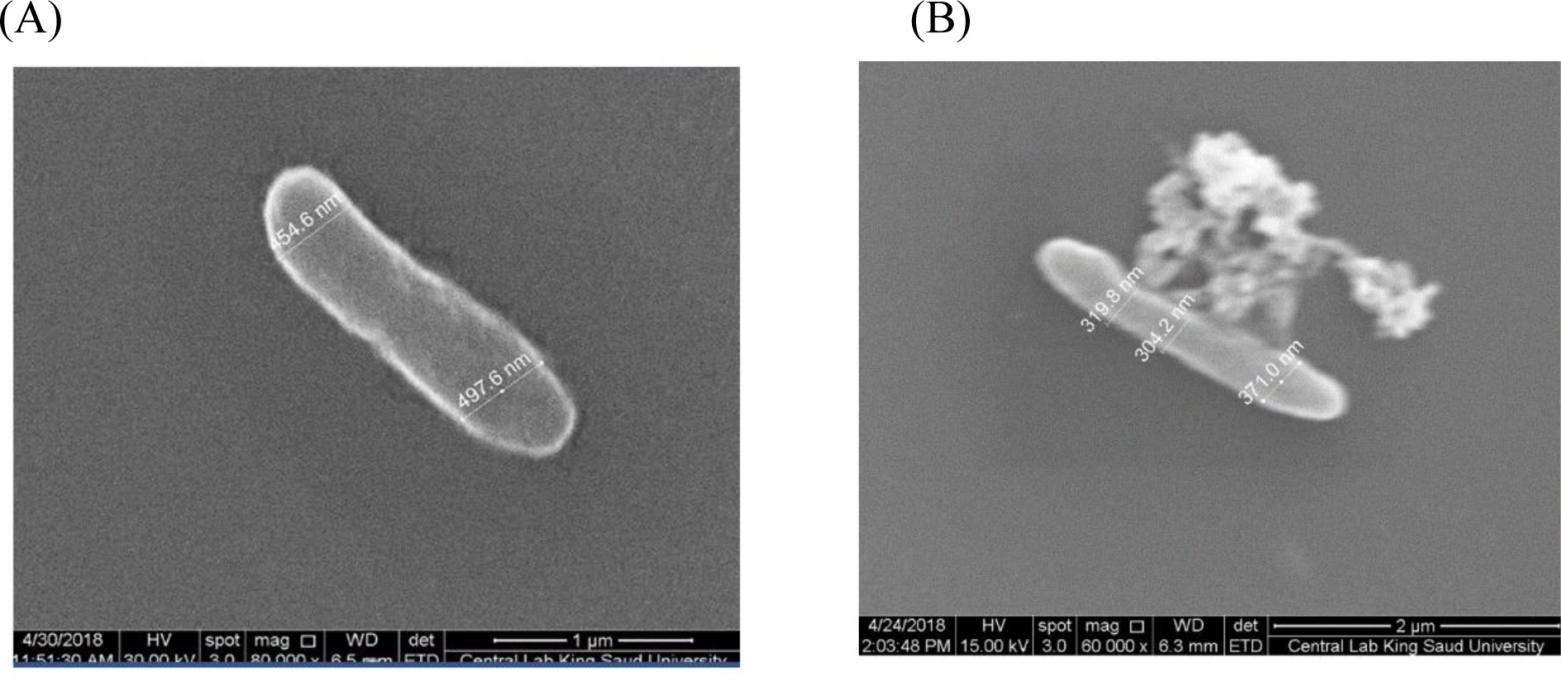
Morphological study of (A) untreated and (B) treated *Escherichia coli* by (IONPs) using SEM.

The antimicrobial property of the IONPs was assessed against three fungi and six bacterial pathogens (two gram-positive and four gram-negative) (Tables 1 and 2 and Fig 5A–5C). IONPs had moderate antimicrobial properties with five pathogens, one fungal (*A*. *niger*), one gram-positive (*Staphylococcus* sp.) and three gram-negative (*Shigella* sp., *Ps*. *aeruginosa*, *E*. *coli*), with inhibition zones ranging from 5 mm to 11 mm (Table 1). SEM was revealed IONPs’ direct binding to the cell wall of *E*. *coli* (Fig 6). The inhibition zone for antimicrobial activity against plant extract ranged from 7 mm in *Staphylococcus* sp. to 8 mm in *Pseudomonas aeruginosa* and *Escherichia coli*, and against Fe ions ranging from 1.1 mm in *Aspergillus niger* to 1.8 mm in *Staphylococcus* sp. and *Klebsiella pneumonia*. NPs and Fe ions are significantly correlated with a correlation coefficient of 0.7266266 and p-value of 0.02659 (Table 2). NPs and plant are significantly correlated with a correlation coefficient of 0.8615747 and p-value of 0.00283. Fe ions, and Plant are not significantly correlated with a correlation coefficient of 0.5689738 and p-value of 0.1099 (Table 2).

**Table 2:** The table output displays the correlation results between NPs, Fe ions and plant.

	NPs	Fe ions	Plant
**NPs**	1.0000000	0.7266266	0.8615747
**Fe ions**	0.7266266	1.0000000	0.5689738
**Plant**	0.8615747	0.5689738	1.0000000

The p-value of the test is 0.02659 which is less than the significance level alpha = 0.05. We can conclude that NPs and Fe ions are significantly correlated with a correlation coefficient of 0.7266266 and p-value of 0.02659. The p-value of the test is 0.00283 which is less than the significance level alpha = 0.05. We can conclude that NPs and plant are significantly correlated with a correlation coefficient of 0.8615747 and p-value of 0.00283. The p-value of the test is 0.1099 which is greater than the significance level alpha = 0.05. We can conclude that Fe ions, and Plant are not significantly correlated with a correlation coefficient of 0.5689738 and p-value of 0.1099.

Untreated *E*. *coli* cells prepared for SEM micrographs were longer, wider, and had a smooth and uninjured surface (Fig 6A). When the cells were treated with NPs (Fig 6B), the surface appeared corrugated, with some dimples, a shorter average length, and a narrower diameter width. The diameter of the width decreased from 454.6 and 497.6 nm to 319.8 and 371 nm, respectively.

## Discussion

### Morphology and characters of biogenic IONPs

An environmentally friendly procedure was employed in this research to produce iron oxide nanoparticles (γ-Fe_2_O_3_). IONPs (Fe_3_O_4_) created by combining (2:1, FeCl_2_: FeCl_3_) and *S*. *costus* root as a reducing agent caused a dark brown color of the mixture, which had previously been yellow. The dark brown colour indicates iron oxide formation, as reported in several studies, including IONPs generation via controlled bio-precipitation from *Mansoa alliacea* leaf extract [31], hydrothermal synthesis of IONPs from *Aloe vera* [32], and plants were used to create iron-polyphenol nanoparticles [33]. The stimulation of exterior Plasmon tremolos in the iron oxide nanoparticles causes the mixture’s color to change, which is a property of the nanoparticles [34]. When an electromagnetic field in the visual range is combined with the collective oscillations of conduction electrons, surface Plasmon vibrations occur as a result of the dipole oscillation raising [35].

Moreover, biogenic IONPs prepared from *S*. *costus* roots peel with spherical, sided, elongated spherical, and prismatic curve-shaped particles with a diameter in the range from 100 to 300 nm, with a mean of 295 nm, with positive potential. The nanoparticles’ zeta potential indicates their stability in the medium in which they are diffused. The particle is considered to be stable if its potential is between +30 and -30 mV. Positively charged and neutral IONPs reduced *S*. *mutans* biofilms more effectively than negatively charged IONPs [36]. The average size of IONPs was 239 nm [37]. Other studies discovered that IONPs prepared from *Platanus orientalis* leaf and *Ficus carica* fruit were a spherical with particle diameters of 30–40 nm and 475 nm, respectively [38, 39].

Some elements were identified alongside Fe peaks bands through the spectral scan range (0–5 keV), indicating that large biomolecules, such as enzymes or proteins, were related to the synthesized IONPs. Our findings agree that the existence of protein or enzyme molecules, which was also affirmed using UV-visible spectra with the biosynthesized Ag-NPs [40–42]. These proteins or enzymes may be able to stabilize the EDX outcomes of the synthesized Ag-NPs [42]. Formation of Ag-NPs on *A*. *terreus* supernatants confirmed the involvement of NADP-dependent reductases [43]. Our findings indicated that the produced IONPs from *S*. *costus* root were created via enzymatic reduction.

### The antimicrobial property of the IONPs

Nanoparticles had greater antimicrobial properties than plant extract and Fe ions against gram-positive, gram-negative bacteria and fungi. The zone of inhibition was greatest against gram-negative *Pseudomonas aeruginosa* (11 mm), followed by gram-positive; *Staphylococcus sp*. (9 mm), gram-negative; *Escherichia coli* (9 mm), and *Shigella* sp. (6 mm), as well as fungi; *Aspergillus niger* (5 mm). The antimicrobial property of IONPs could be linked to the oxidative stress system caused by reactive oxygen species (ROS), which could destroy biomolecules like DNA and proteins [44, 45]. Following IONPs diffusion within the culture media, various oxide reduction reactions involving both the species present in magnetite, Fe3+, and Fe2+, are followed, resulting in the generation of different and more potent reactive oxygen species [46]. A number of factors influence the antimicrobial properties of IONPs. In some studies, both gram-positive and gram-negative bacteria are susceptible to the antibacterial property of IONPs [26]. After being synthesized with *P*. *guajava* leaf extract, hematite nanoparticles were discovered to be powerful antibacterial components against Gram-positive and Gram-negative bacteria [47]. When checked on bacterial pathogens, the antibacterial action of IONPs derived from *L*. *siceraria* was found to be effective against *A*. *hydrophila* (gram-positive) and *E*. *coli* (gram-negative) [48]. The most susceptible bacteria to IONPs of green synthesized by the disc diffusion method were four (*S*. *typhi*, *S*. *aureus*, *S*. *enterica*, and *K*. *pneumonia*) out of nine [49]. Hematite nanoparticles have previously been evaluated for antifungal activity, with the greatest inhibition zone recognized against *P*. *chrysogenum* (28.67 mm), accompanied by *A*. *niger* (26.33 mm), *T*. *roseum* (22.67 mm), and *A*. *alternata* (21.33 mm) [50]. The antimicrobial effect of the prepared nanoparticles against Gram-positive bacterium *L*. *monocytogenes* (12 mm) and two fungi, *A*. *flavus* (13 mm) and *P*. *spinulosum* (14 mm) was moderate [51].

The IONPs have antimicrobial properties [25]. Both gram-positive and gram-negative bacteria are resistant to IONPs [26]. Various studies on the eco-friendly creation of IONPs were conducted utilizing various plant parts to assess their bioactivity, IONPs synthesized using *Couroupita guianensis* fruit extract had effective bactericidal against tested human pathogens [27]. IONPs derived from corn plant extract displayed antibacterial activity against the tested bacterial species [24].

Bacterial cells were unharmed in the absence of IONPs, but after NP treatment, the vast majority of cells had reduced diameters and various stages of membrane injury, with some lysed. Electron microscopy revealed that IONPs can attach directly to the cell wall of *E*. *coli*, as well as invade and concentrate in the cytoplasm, causing vacuole creation and cell wall alterations [52, 53]. IONPs can impair bacterial cell wall integrity [54]. Antibiotic-resistance genes in antibiotic-resistant bacteria found in hospitals can be limited by IONPs [15]. Bacteria dissociate from a cell membrane due to magnetic and paramagnetic IONP properties, which cause cell membrane damage, membrane explosion, cell fusion, and death [55].

## Conclusion

We described the production of iron oxide nanoparticles (γ-Fe_2_O_3_) from an aqueous extract of *S*. *costus* root and a combination of (2:1, FeCl_2_: FeCl_3_). The synthetic IONPs had a positive potential and were spherical and prismatic curve-shaped, with a mean size of 295 nm. *S*. *costus* contains the natural substances that are in charge of the bio-reduction mechanism. Antimicrobial activities of IONPs were discovered against gram-negative *P*. *aeruginosa*, *E*. *coli*, and *Shigella* sp., as well as gram-positive *Staphylococcus* sp. and fungi such as *A*. *niger*. The creation of iron oxide nanoparticles from *S*. *costus* root is an eco-friendly, simple, effective, and low-cost biological method that may be recognized as a strong antibacterial substance. More focus and investigation are currently needed to control the size and shape of nanoparticles and determine the exact mechanism of IONP formation by green plants.
